# Antioxidant Properties and Geroprotective Potential of Wheat Bran Extracts with Increased Content of Anthocyanins

**DOI:** 10.3390/antiox12112010

**Published:** 2023-11-17

**Authors:** Daria V. Mikhailova, Oksana G. Shevchenko, Denis A. Golubev, Elena Y. Platonova, Nadezhda V. Zemskaya, Olesya Yu. Shoeva, Elena I. Gordeeva, Sergey A. Patov, Mikhail V. Shaposhnikov, Elena K. Khlestkina, Alexey Moskalev

**Affiliations:** 1 Institute of Biology of Komi Scientific Centre of the Ural Branch of the RAS, 167982 Syktyvkar, Russia; 2 Federal Research Center N. I. Vavilov All-Russian Institute of Plant Genetic Resources (VIR), 190031 St. Petersburg, Russia; 3 Institute of Cytology and Genetics of the Siberian Branch of Russian Academy of Sciences (ICG SB RAS), 630090 Novosibirsk, Russia; 4 Institute of Chemistry of Komi Scientific Centre of the Ural Branch of the RAS, 167000 Syktyvkar, Russia

**Keywords:** antioxidants, anthocyanins, wheat, *Drosophila*, geroprotector

## Abstract

In recent years, there has been a focus on breeding wheat with high anthocyanin levels in order to improve food quality and human health. The objective of this study was to examine the antioxidant and geroprotective properties of wheat bran extracts using both in vitro and in vivo research methods. Two wheat lines were used: one with uncolored pericarp (anthocyanin-free) and another with colored pericarp (anthocyanin-containing). These lines differed in a specific region of chromosome 2A containing the *Pp3/TaMyc1* gene, which regulates anthocyanin production. High-performance liquid chromatography-mass spectrometry revealed the presence of cyanidin glucoside and cyanidin arabinoside in the anthocyanin-containing wheat bran extract (+AWBE), while no anthocyanins were found in the anthocyanin-free wheat bran extract (−AWBE). The +AWBE showed higher radical scavenging activity (DPPH and ABTS assays) and membrane protective activity (AAPH oxidative hemolysis model) compared to the −AWBE. Both extracts extended the lifespan of female *Drosophila*, indicating geroprotective properties. This study demonstrates that wheat bran extracts with high anthocyanin levels have antioxidant and geroprotective effects. However, other secondary metabolites in wheat bran can also contribute to its antioxidant and geroprotective potential.

## 1. Introduction

According to the free radical theory of aging, oxidative stress caused by both endogenous and exogenous free radicals accelerates the aging process by damaging sensitive cellular targets [1,2]. The free radical theory suggests that antioxidants can reduce the level of damage to cellular structures, thereby delaying age-related diseases and slowing down aging [3,4]. Anthocyanins are of particular interest as antioxidants that are constantly supplied to the body, due to their high content in numerous plant-based foods [5]. Anthocyanins belong to the flavonoids group of polyphenols. These water-soluble pigments are responsible for a variety of the red, blue, and purple colors of many berries, fruits, vegetables, legumes, and grains [6,7].

Experimental research indicates that anthocyanins possess the ability to scavenge or neutralize free radicals [5], chelate metals [8], impact signaling pathways [9], decrease the level of pro-inflammatory markers [10], mitigate the risk of obesity [11], cardiovascular pathologies [12], cancer [13], diabetes [14], and neurodegeneration [15,16].

*Drosophila melanogaster* serves as a well-established model to investigate the mechanisms underlying aging and to identify geroprotective compounds [17,18]. This species combines extensive knowledge of the taxonomy, physiology, genetic basis, and biological mechanisms of aging with a short life span, as well as the ability to control housing conditions and ease of cultivation [18,19]. Furthermore, the diet of fruit flies can be adjusted significantly, allowing for the regulation of nutrient and additive intakes [20,21,22]. Previously, in a number of studies, the antioxidant and geroprotective properties of anthocyanins were studied using the *D. melanogaster* model [23]. For example, anthocyanin extract from cranberry (*Vaccinium macrocarpon* L.) was shown to increase the average lifespan of male flies by 10% and also reduce the mortality rate caused by treatment with hydrogen peroxide (H_2_O_2_) [24]. In research by our group, it was previously established that anthocyanin extracts from the fruits of chokeberry (×*Sorbaronia mitschurinii*) [25], honeysuckle *(Lonicera caerulea* L.) [26], and rowan (*Sorbus aucuparia* L.) [27] exhibit antioxidant activity and contribute to an increase in the lifespan of *D. melanogaster*. This data suggests that anthocyanins possess geroprotective potential.

However, it is worth noting that free radicals play a crucial physiological role within cells. They participate in the regulation of the redox potential [28,29] and in the transmission of intracellular signals, which are critical for the organism’s lifespan, including gene expression, apoptosis, cell growth, and cell cycle [30,31]. Thus, an organism’s lifespan relies on maintaining a fine equilibrium between the opposing impacts of free radicals [1,3,32]. Whether reducing oxidative damage below normal levels is sufficient to increase life span remains an open inquiry.

Some colored varieties of wheat (*Triticum aestivum* L.) accumulate anthocyanins and, along with berries and fruits, can serve as an important food source [33]. In wheat grains, anthocyanin pigments accumulate in the pericarp tissue or aleurone layer, resulting in a purple or blue color [34,35].

Ma et al. [36] provides a comprehensive review of the current data on polyphenolic composition in wheat varieties with diverse grain color parameters. Phenolic acids and flavonoids are the predominant phenolics in wheat grains [36]. Phenolic acids, including gallic, protocatechuic syringic, and vanillic in the hydroxybenzoic group, and ferulic, caffeic, chlorogenic, sinapic, and *p*-coumaric in the hydroxycinnamic group, and flavonoids including catechin and epicatechin, have been identified in the bran of various white wheat varieties (Avalon, C-306, HS-490, HD-3086, and HD-2967) using high-performance liquid chromatography (HPLC) [37,38,39]. Comparable phenolic acids were also detected in different fractions of red wheat bran [39]. The accumulation of phenolic compounds within the seed coat results in a darkening of its color [36,39]. For example, in the bran of purple wheat CDC Primepurple, a total of 13 anthocyanin compounds were detected, with the five most abundant being cyanidin-3-(6-malonyl glucoside), cyanidin-3-glucoside, cyanidin-3-rutinoside, peonidin-3-glucoside, and peonidin-3-(6-malonylglucoside) [40].

The high antioxidant activity of anthocyanins implies that colored wheat varieties and different fractions of their grain have the potential to improve the physiological state of a living organism [16,33,41]. The purpose of this work was to study the antioxidant activity (in vitro) of bran extracts of two isogenic wheat lines, differing in the content of anthocyanins in the pericarp of the grain, as well as to comparatively assess their effect on the stress resistance and lifespan of *Drosophila melanogaster.*

We investigated wheat bran extract as a natural source of anthocyanins, which can be regularly consumed by incorporating purple grain into bread preparation without any additional additives or supplements [42]. Previous studies have demonstrated that anthocyanins remain intact during bread production [43,44]. The antioxidant activity of these bioactive compounds was tested using varied in vitro systems such as mammalian erythrocytes and substrates based on tissue homogenates of laboratory animals. To study the effects of wheat bran extracts on the whole organism level, *D. melanogaster* was used as a model, which, in recent years, has been increasingly applied in studies of nutrition in aging and longevity [18,20,21,22]. We were the first to reveal the geroprotective and adaptogenic effects of wheat bran-derived anthocyanin extracts on the motility and survival rates of *D. melanogaster* under both normal and stressful conditions. The findings of our study may contribute to the development of strategies aimed at promoting healthy longevity and enhancing the quality of human life via healthy and functional nutrition.

## 2. Materials and Methods

### 2.1. Plant Material

Two isogenic wheat lines were tested: anthocyanin-free (i:S29*Pp-A1Pp-D1pp3^P^*) and anthocyanin-containing (i:S29*Pp-A1Pp-D1Pp3^P^*), which differ from each other in a small region of chromosome 2A containing the *Pp3/TaMyc1* gene, which regulates the biosynthesis of anthocyanins in the pericarp grains. Both lines were bred on the basis of the red grain wheat variety Saratovskaya 29 [45]. Wheat lines were cultivated in the Novosibirsk region at the experimental breeding and genetic complex of the Institute of Cytology and Genetics SB RAS (55°02′ N, 82°56′ E) during the year 2018.

To obtain bran, the wheat grain was hydrated to a 15.5% moisture content for 20 h prior to milling. The amount of water required for hydration was calculated based on the initial moisture content and grain hardness. The grain was ground with a laboratory roller mill MLV-1 (COKB, Russia), resulting in 70% single-grade flour and bran. The flour was sifted using a sieve (silk sieve No. 25 with cell size 0.3 × 0.3 mm), and only the bran particles that were larger than this size were collected for anthocyanin extraction. Throughout the experiment, the bran was stored in sealed bags at +4 °C in a dry, dark place. Fresh extracts were prepared as required for analysis.

### 2.2. Extraction

Extraction of anthocyanins from wheat was carried out according to the previously described method [43]. To obtain the extract, bran was mixed with a 1% aqueous solution of hydrochloric acid (HCl) in the proportion of 1 g of bran per 10 mL of HCl. Next, the resultant mixture was stirred on a magnetic stir plate for one hour at 37 °C and under incubation conditions within a dry-air thermostat TS-1/20 SPU (JSC Smolensk SKTB SPU, Smolensk, Russia). Then, the mixture was centrifuged at 5000 rpm (2500× *g*) for 15 min at a temperature of +4 °C using an Eppendorf 5430R refrigerated centrifuge (Eppendorf, Hamburg, Germany). The resulting extract (supernatant) was used to treat experimental flies.

### 2.3. High Performance Liquid Chromatography-Mass Spectrometry

To prepare samples for high-performance liquid chromatography-mass spectrometry (HPLC-MS) analysis, 1 mL of the extract was dissolved in 10 mL of distilled water and applied to a previously prepared Hypersep C18 microcolumn (Thermo Electron Corporation, Waltham, MA, USA, 100 mg/1 mL/100 pkg), where the column was then washed with 10 mL of distilled water. Anthocyanins from the column were washed off with 1 mL of acetonitrile solution in water (10% formic acid) in a 1:1 (*v*/*v*) ratio. The resulting solution was analyzed via HPLC-MS.

To study the extract samples, we used a Thermo Finnigan Surveyor high-performance liquid chromatography-mass spectrometry (HPLC-MS) system equipped with a photodiode array (PDA) detector capable of scanning the ultraviolet-visible wavelength range from 200 to 600 nm and a mass selective detector (Thermo Fisher Scientific Inc., Waltham, MA, USA). The analysis was carried out on an ODS Hypersil C 18 column (100 mm × 2.1 mm, 5 μm). Aqueous (H_2_O) solutions of acetonitrile (AcN) and 10% formic acid (HCOOH) were used as eluents. The analysis used a gradient mode of eluent A (10% HCOOH + 90% H_2_O) and B (10% HCOOH + 40% H_2_O + 50% AcN): 12% B at 1 min, 30% B at 26 min, 100% B at 35 min, 100% B at 38 min, 12% B at 43 min, and 12% B at 50 min. The eluent flow rate is 1 mL/min, the column temperature is +40 °C, and the injected sample volume is 5 µL. Detection was carried out at a wavelength of 520 nm, for anthocyanin-containing wheat bran extract (+AWBE), and 340 nm, for anthocyanin-free wheat bran extract (−AWBE).

### 2.4. Analysis of Anthocyanins

To perform the analysis, 100 mg of the extract was dissolved in 10–15 mL of distilled water acidified with acetic acid (10%) and applied to a column filled with a reverse-phase sorbent (Diasorb 130 C 16T). The sorbent was preliminarily washed with 200 mL of acidified water. After applying the substance to the column, it was washed again with 200 mL of acidified water to remove any accompanying sugars and soluble salts. Then, the target substances from the column were washed off with solutions of acetonitrile in acidified water with 4–20% *v/v* (volume percent) in steps of 2%. The approximate volume until the complete release of each individual substance was 200 mL. Substances from the column were collected into 10 mL vials and analyzed via HPLC. Eluates containing anthocyanins and having the same chromatographic mobility were combined, evaporated, and dried under vacuum at low temperature until a constant weight was achieved, removing any water or crystallization. Purified and dried anthocyanins were analyzed for their structure using ^13^C and ^1^H NMR methods, along with HSQC, HMBC experiments, and mass spectrometry.

The NMR spectrum of cyanidin Glu ((2-(3,4-dihydroxyphenyl)chromenylium-3,5,7-triol, (D_2_O, δ, ppm)) is as follows: in the ^1^H NMR spectrum of the substance, there are characteristic signals at 7.2 ppm (1H, c, H4), 6.7 (1H, t, H6), 6.56 (1H, d, H8), 6.52 (1H, d, H2′), 6.8 (1H, dd, H5′), 6.61 (1H, d, H6′), as well as ^13^C signals 170.0, 150.1, 121.1, 161.5, 127.3, 163.6, 145.0, 136.9, 130.8, 105.6, 120.6, 164.6, 162.4, 130.9, and 130.6, which confirms the pyran skeleton of the substance, while the hydroxyl groups are located at the 5, 7, 3′, and 4′ positions of the aromatic rings. NMR signals ^1^H 4.78 (1H, d, J = 7.5), ^13^C 100.5 ppm correspond to the β-D-glucose fragment associated with the anthocyanin molecule at position 3C (corresponding to the signal of the carbon atom at 150.1 ppm); m/z 486.21.

Mass spectrometry was conducted at 275 °C with a capillary voltage of 5 kV and a helium flow of 7 L/min, detecting positive ions [M^+^] within the mass range of a 100–2000 mass-to-charge ratio (*m*/*z*). To identify the carbohydrate fragment, acid hydrolysis of cyanidin glucoside was performed. After neutralization, the aglycone was removed from the solution using methyl chloride, of which the remaining carbohydrate fragments in the mother liquor were evaporated to dryness and acylated with acetic anhydride in glacial acetic acid using perchloric acid as a catalyst. The sugar acetates were analyzed via GLC and compared with the retention times of previously analyzed sugar acetates. Similarly, we analyzed the other anthocyanins described in the article.

Pure anthocyanins were analyzed via HPLC to determine their retention times. HPLC was performed according to the previously described conditions. Then, precise amounts of anthocyanins were selected, their calibration solutions were prepared in the concentration range from 0.01–0.1 mg/mL, and a calibration graph was constructed (Figure S1, for example).

### 2.5. Evaluation of Antioxidant Activity

When studying the antioxidant activity of the compounds, we performed no experiments on animals; the analysis was carried out exclusively in vitro. We used the erythrocytes, brain, and testes tissues of intact laboratory mice obtained from the scientific collection of experimental animals at the Institute of Biology of Komi Science Centre of the Ural Branch of the Russian Academy of Sciences, which is also registered as a unique scientific installation within the scientific and technological infrastructure of the Russian Federation http://www.ckp-rf.ru/usu/471933/ (accessed on 15 November 2023). The animals were handled in accordance with the Regulations on the vivarium of experimental animals (protocol No. 1 dated 24 January 2017) considering sanitary-hygienic and bioethical aspects. The permission of the Ethical Committee was not necessary.

The incubation of erythrocytes, as well as the substrates based on tissue homogenates of laboratory animals, was carried out in an orbital shaker-incubator Biosan ES-20 (Biosan, Rīga, Latvia). Absorbance was measured using a Thermo Spectronic Genesys 20 spectrophotometer (Thermo Fisher Scientific Inc., Waltham, MA, USA). Each experiment was performed in 4–6 replicates.

Radical scavenging activity (RSA) of the extracts was evaluated by measuring their ability to interact with DPPH (2,2-diphenyl-1-picrylhydrazyl) and ABTS (2,2′-azino-bis-(3-ethylbenzthiozoline-6-sulfonic acid) diammonium salt) using established procedures with minor modifications [46,47,48,49]. Both tests are widely used to evaluate the antioxidant activity of pure compounds and various extracts.

In the DPPH assay, the stock solutions of the analyzed extracts were added to a DPPH solution in MeOH (1% *v*/*v*). Each mixture was then shaken vigorously and kept in the dark at room temperature for 30 min. The absorption decrease was measured at λ 517 nm. The RSA was calculated as a percentage of DPPH discoloration using the following formula: RSA/% = 100 × (1 − At/Ac), where At is the absorbance of the sample containing the test extracts and Ac is the absorbance of the control sample with all reagents except for the extracts. The Trolox solution (6-hydroxy-2,5,7,8-tetramethylchroman-2-carboxylic acid) served as a reference compound.

In the ABTS assay, ABTS^•+^ was produced by reacting the ABTS stock solution (7 mM) with potassium persulfate (2.45 mM) and allowing the mixture to stand in the dark at room temperature for 12–16 h before use. The ABTS radical cation solution was diluted with an ethanol to an absorbance of 0.70 at 734 nm. The stock solutions of the studied extracts were added to the ABTS radical cation solution (1% *v*/*v*). Then, each mixture was shaken vigorously and kept in the dark at room temperature for 30 min. The absorption decrease was measured at λ 734 nm. The RSA was calculated as a percentage of ABTS discoloration using the following equation: RSA/% = 100 × (1 − At/Ac), where At is the absorbance of the sample containing the test extracts and Ac is the absorbance of the control sample with all reagents except for the extracts. The Trolox solution served as a reference compound.

The antioxidant activity (AOA) of the extracts was evaluated by the ability to inhibit the accumulation of TBA-reactive substances (substances that react with 2-thiobrabituric acid, TBA-RS), Fe^2+^/ascorbate-, or H_2_O_2_-initiated lipid peroxidation (LPO) in the substrate (oil in water emulsion) that was obtained from tissue homogenates (brain and testes) of laboratory mice [50,51,52,53]. The brain or testes were homogenized in physiological saline (pH 7.4) (10% *v*/*v*) and centrifuged at 3000 rpm (1600× *g*) for 10 min using the CM-6M centrifuge (ELMI, Rīga, Latvia). The low-speed supernatant was separated. The stock solutions of extracts were added to the supernatant at final concentrations of 0.05 and 0.5 mg/mL (1% *v*/*v*); then, in 30 min, LPO was initiated by adding a freshly prepared solution of FeCl_2_ and ascorbic acid (brain homogenate) or H_2_O_2_ (testes homogenate). The samples were stirred gently at 37 °C for 1 h (brain homogenate) or 3 h (testes homogenate), and then the reaction was stopped by adding trichloroacetic acid (20%) and 2-thiobarbituric acid (0.7%) to the substrate. The reaction mixture was heated in a boiling water bath for 15 min. After cooling, the precipitate was removed via centrifugation (3000 rpm (1600× *g*), 10 min). The concentration of secondary lipid peroxidation products reacting with 2-thiobarbituric acid (TBA-RS) was determined at λ 532 nm using the extinction coefficient of 1.56 × 10^5^ M^−1^cm^−1^ [53,54,55]. The Trolox solution served as a reference compound.

To analyze the extracts’ erythrotoxicity, antioxidant properties, and membrane-protective activity, we used a 0.5% (*v*/*v*) suspension of laboratory mice erythrocytes in phosphate-buffered saline (PBS, pH 7.4). Erythrotoxicity was evaluated by the extracts’ ability to induce erythrocyte hemolysis within 5 h of incubation. The antioxidant and membrane-protective activities of the extracts were determined by the degree of inhibition of oxidative hemolysis induced by 2,2′-azobis(amidinopropane) dihydrochloride (AAPH) or H_2_O_2_ [56,57,58]. The Trolox solution served as a reference compound.

### 2.6. Drosophila Line and Maintenance Conditions

The wild-type *D. melanogaster* line *Canton-S* used in this study was obtained from the Bloomington *Drosophila* Stock Center at Indiana University (#64349, Bloomington, IN, USA). The flies were maintained in a constant climate chamber Binder KBF720-ICH (Binder, Tuttlingen, Germany) at a temperature of 25 °C, a relative humidity of 60%, and a 12 h light/12 h dark lighting cycle. The nutrient medium consisted of the following components: water—1 L, semolina—30 g, dry yeast—8 g, sucrose—30 g, and agar agar—7 g, with the addition of 5 mL of a 10% ethanolic solution of nipagin (methyl 4-hydroxybenzoate, Merck, Rahway, NJ, USA) and 5 mL of propionic acid (Merck, Rahway, NJ, USA). Male and female flies were kept in distinct vials, each with up to 30 individuals.

*Drosophila* imagoes were used in the experiments since the effects of nutrition are more prolonged and this stage is suitable for lifespan studies [22]. Additionally, *Drosophila* imago has organs and tissues that perform comparable functions to those of most mammalian organs (heart, kidney, digestive system, nervous system, muscular system, reproductive system, and adipose tissue), allowing for the study of functional aging [19,21], such as alterations in locomotor activity, which are linked to the aging of the muscular system [59,60].

### 2.7. Lifespan Analysis and Treatment

Control and experimental individuals were collected within 24 h after imago hatching. Using a Flowbuddy carbon dioxide anesthesia apparatus (Genesee Scientific, El Cajon, CA, USA), flies were sorted by sex and placed in vials of 30 individuals. Starting from the first day of life of the imago, the number of dead individuals was counted daily, and the flies were transferred to a fresh medium twice a week.

Wheat extracts at a concentration of 0.1 g/mL were applied to the surface of the food medium in a volume of 30 μL per vial. As a control, 30 μL of a 1% aqueous HCl solution was applied to the medium. The treatment was carried out throughout their lifetime. For each group, 120–150 individuals were used. The experiments were carried out in four biological replicates.

### 2.8. Analysis of Stress Resistance

After imago hatching, the flies were separated by sex and treated with the extracts at a concentration of 0.1 g/mL for 10 days. To assess stress resistance, flies were individually placed in glass capillaries 60 mm in length and 5 mm in diameter, filled with a nutrient medium consisting of 2% agar and 5% sucrose. Throughout the experiment, locomotor activity was analyzed using the *Drosophila* Activity Monitor DAM (Trikinetics Inc., Waltham, MA, USA). The locomotor activity data from individual flies were pooled into 60 min periods and analyzed. The time of death of individual flies was determined by the complete absence of locomotor activity.

To study resistance to oxidative stress, paraquat (#856177, Merck, Darmstadt, Germany) was added to the nutrient medium at a concentration of 20 mM. When assessing resistance to starvation, flies were kept on a 2% agar medium without the addition of sucrose. Hyperthermia was induced by continuously exposing flies to a temperature of 35 °C. Stressful conditions were applied until the flies died without being transplanted into fresh capillaries. For each experimental variant, 32 males and 32 females were analyzed. Each experiment was repeated at least two times.

### 2.9. Analysis of Locomotor Activity

Assessment of age-dependent changes in the locomotor activity of *Drosophila* was carried out once a week, from 2 to 9 weeks of age, using the Locomotor Activity Monitor LAM25 (TriKinetics Inc., Waltham, MA, USA). Flies were kept in vials with improved transparency (#32–118, Genesee Scientific, El Cajon, CA, USA), in the amount of 10 individuals of the same sex per vial. The data from the locomotor activity monitor (the total number of activations of infrared motion sensors) were recorded for 24 h and presented as the average daily activity per individual. For each group, five vials were used, and each vial was considered as a replication of the experiment.

### 2.10. Statistical Analysis

The statistical significance of differences in parameters characterizing the antioxidant activity of the extract was assessed using the Mann–Whitney test. To assess the significance of differences between survival curves, the log-rank test with Bonferroni correction for multiple comparisons was used [61]. To assess the statistical significance of differences in a median and maximum life span, Fisher’s exact test with Bonferroni correction was used. To analyze the statistical significance of differences in locomotor activity depending on age and treatment, two-factor analysis of variance (ANOVA) was used, followed by pairwise comparison of variants using a post hoc Tukey test. Data visualization and processing were performed using software packages TIBCO Statistica version 13.3 (TIBCO Software, Palo Alto, CA, USA), Excel 2010 (Microsoft, New York, NY, USA), and online application for survival analysis OASIS 2 [62].

## 3. Results

### 3.1. Chemical Analysis of Wheat Extracts

**Figure 1:**
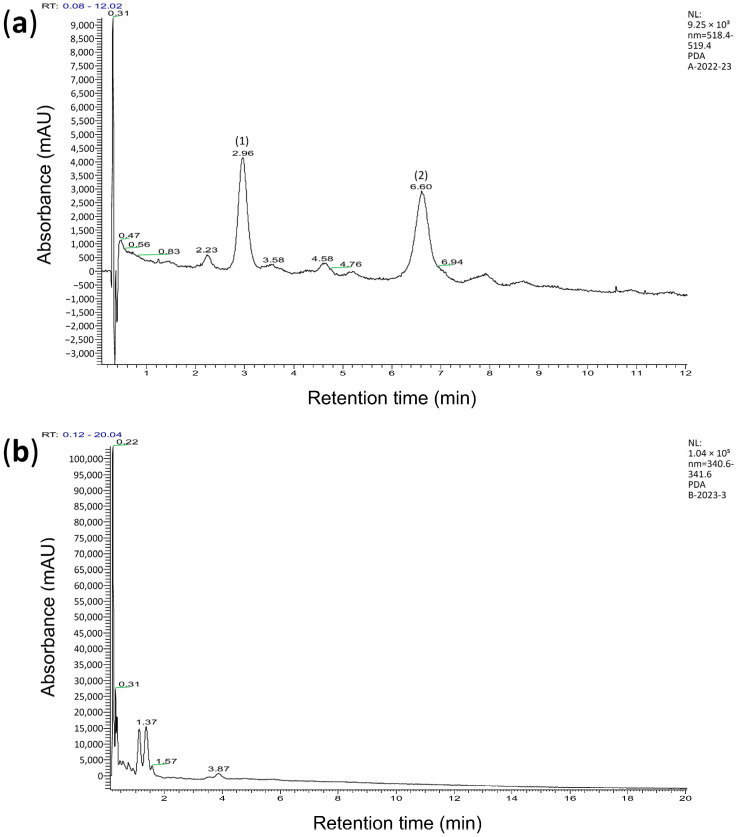
Chromatogram showing the content of hydrochloric acid extract of brans from anthocyanin-containing (**a**) and anthocyanin-free (**b**) wheat lines. (**a**)—two anthocyanins are presented: (1) cyanidin glucoside with a retention time of 2.96 min, and (2) cyanidin arabinoside with a retention time of 6.60 min. (**b**)—anthocyanins are not observed.

The chromatogram of wheat extracts was obtained via high-performance liquid chromatography with mass spectrometry (HPLC-MS). The retention times of known substances and their molecular ions were used to identify the substances. The study found that +AWBE contains two major substances, cyanidin glucoside (*m*/*z* 486.21) and cyanidin arabinoside (*m*/*z* 426.33), as well as several minor anthocyanins (Figure 1a, Table S1). No anthocyanins were found in the −AWBE (Figure 1b).

### 3.2. Antioxidant Activity of Extracts

The ability of various compounds to inhibit free radical oxidation is influenced by many factors and cannot be correctly assessed using one universal method [46,63,64,65]. Therefore, the assessment of the antioxidant activity (AOA) of extracts was carried out using several in vitro methods, which we used in previous studies of plant extracts [26,27,66].

Table 1 shows that the radical scavenging activity (RSA) of the +AWBE was higher in both the stable radical DPPH test (*p* = 0.021) and the more active radical cation ABTS^•+^ test (*p* = 0.021) compared to the −AWBE. At a concentration of 0.5 mg/mL, the +AWBE was slightly less effective than Trolox in the ABTS test.

At concentrations of 0.05 and 0.5 mg/mL, both extracts almost completely inhibited (*p* = 0.021) both spontaneous and Fe^2+^/ascorbate-initiated LPO in a substrate based on the brain homogenate of laboratory animals, which follows from the decrease in the concentration of secondary LPO products that react with 2-thiobarbituric acid (TBA-RS) (Table 1). In this system, both extracts were practically as effective as Trolox in inhibiting LPO. Bran extracts from both wheat lines exhibited effective inhibition of H_2_O_2_-induced LPO in a substrate based on the testes of laboratory animals (Table 1).

Before investigating the antioxidant properties of wheat extracts on erythrocytes subjected to oxidative hemolysis, it was necessary to confirm the absence of significant erythrotoxicity from the extracts. According to the results of preliminary experiments, the level of hemolysis of intact erythrocytes after 5 h of incubation was 3.2 ± 0.1%, and in the presence of the studied extracts at a concentration of 0.05 mg/mL, it was no more than 7.8 ± 0.1%, which does not prevent further studying their biological activity using erythrocytes from the blood of laboratory animals as a test object.

Under conditions of AAPH-induced oxidative hemolysis, both extracts showed statistically significant membrane protective activity (*p* = 0.004). This was evidenced via a reduction in the intensity of oxidative hemolysis in the presence of the extracts when compared to the controls (Table 2). However, cell survival under the influence of peroxyl radicals generated during the thermal decomposition of AAPH in the presence of +AWBE was significantly higher than under the influence of −AWBE (*p* = 0.009). The protective effect of +AWBE persisted throughout the entire 5 h experiment, while the protective effect of −AWBE only lasted for 4 h.

Under conditions of H_2_O_2_-induced oxidative hemolysis, the bran extracts of both wheat lines also had statistically significant (*p* = 0.004) membrane protective activity. At the same time, there were no statistically significant differences in cell survival in the presence of wheat bran extracts from different lines under the influence of H_2_O_2_. The obtained data suggest that the antioxidant activity of the extracts in this experiment is not related to the presence of anthocyanins, but is due to the presence of other compounds.

### 3.3. Effects of Bran Extracts on Drosophila Lifespan

To determine the relationship between the antioxidant properties and the potential geroprotective activity of bran extracts, we evaluated differences in the lifespan of *D. melanogaster* in the control (1% HCl solution) and extract-treated (0.1 g/mL of +AWBE or 0.1 g/mL of −AWBE) groups, as well as between groups treated with extracts from different wheat bran.

**Figure 2:**
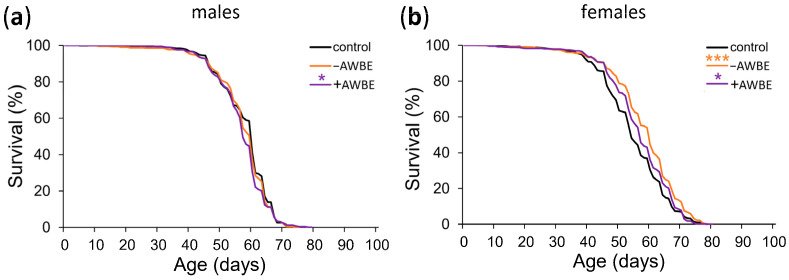
Effect of −AWBE and +AWBE on the lifespan of *D. melanogaster* males (**a**) and females (**b**). * *p* < 0.05, *** *p* < 0.001—log-rank test with Bonferroni correction.

Compared to the control, +AWBE decreased the median lifespan of males by 5%, *p* < 0.001 (Table 3, Figure 2a), but increased it of females by 6%, *p* < 0.001 (Table 3, Figure 2b). When females were treated with −AWBE, the median lifespan increased by 11% (*p* < 0.001) and the maximum lifespan increased by 6% (*p* < 0.01) compared to the control. It is noteworthy that the effects of −AWBE were statistically and significantly higher than the effects of +AWBE on the median and maximum lifespan of females by 5% and 4%, respectively (*p* < 0.001) (Table 3, Figure 2b). The obtained results show that wheat bran extracts have a geroprotective effect. The −AWBE had the most pronounced effect on lifespan. The effect on lifespan varied by sex. At the same time, the geroprotective effect was observed only in females.

### 3.4. Age Dynamics of Locomotor Activity

**Figure 3:**
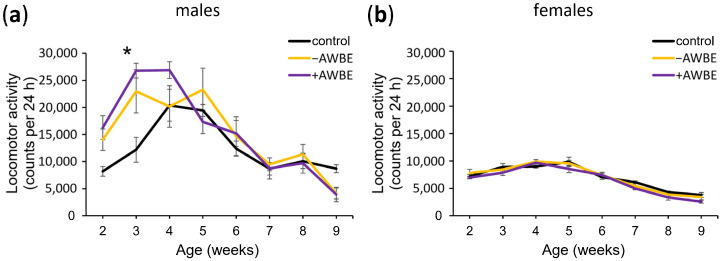
The influence of −AWBE and +AWBE on the age-related dynamics of locomotor activity of *D. melanogaster* males (**a**) and females (**b**). * *p* < 0.01, two-way ANOVA with Tukey’s post hoc test. Locomotor activity was measured by calculating the total number of times the infrared motion sensors were triggered in a 24 h period at 6-day intervals. Error bars indicate standard error of the mean.

It has been previously found that the locomotor activity of *D. melanogaster* decreases with age, similar to the progressive decline in locomotor ability observed in humans [59,60]. To identify the effects of −AWBE (0.1 g/mL) and +AWBE (0.1 g/mL) on age-dependent decline in locomotor function, we assessed locomotor activity at different ages. Two-way ANOVA revealed a statistically significant effect of age (*p* < 0.05), treatment (*p* < 0.001), as well as their interaction (*p* < 0.01) on the locomotor activity of males (Table 4). Post hoc analysis showed a statistically significant (*p* < 0.01) increase in the locomotor activity of males treated with +AWBE at the age of three weeks compared to the control individuals (Table 4, Figure 3a). In female flies, both age (*p* < 0.05) and diet (*p* < 0.001) exhibit significant effects; however, their interaction was not statistically significant (*p* > 0.05), and post hoc analysis did not identify any statistically significant differences in the locomotor activity of females at different ages (Table 4, Figure 3b).

Thus, a statistically significant effect of age and treatment on the locomotor activity of male and female flies was established, along with a combined effect of these factors on male locomotor activity. It was found that +AWBE has a significant effect on the locomotor activity of males.

### 3.5. Resistance to Adverse Environmental Factors

We examined the impact of −AWBE (0.1 g/mL) and +AWBE (0.1 g/mL) on the resistance of *D. melanogaster* individuals to unfavorable environmental factors, including treatment with the prooxidant paraquat, starvation, and hyperthermia.

**Figure 4:**
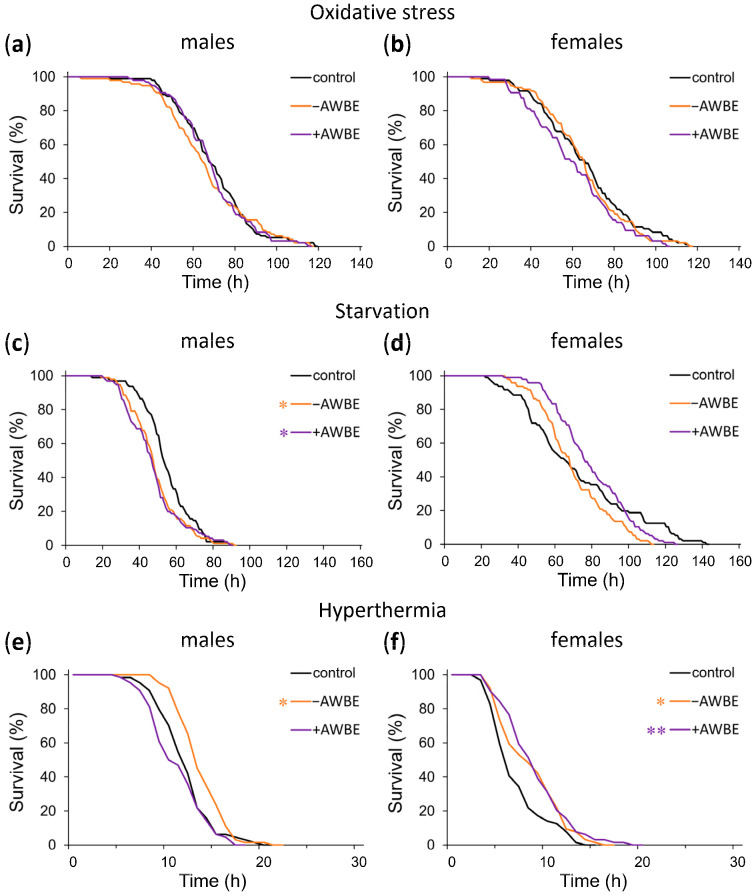
The influence of −AWBE and +AWBE on the resistance to adverse environmental factors of *D. melanogaster* males (**a**,**c**,**e**) and females (**b**,**d**,**f**). * *p* < 0.05, ** *p* < 0.01—log-rank test with Bonferroni correction.

When comparing the effects of treating with −AWBE and +AWBE relative to the control group under conditions of oxidative stress, no statistically significant effects (*p* > 0.05) were observed on the survival time of *Drosophila* males and females (Table 5, Figure 4a,b).

Under conditions of starvation, pre-treatment with −AWBE and +AWBE decreased the median survival time of males by 9% (*p* < 0.01) and 11% (*p* < 0.001), respectively, and a leftward shift of the mortality curves was observed (Table 5, Figure 4c). Additionally, in females, treatment with −AWBE caused a 19% (*p* < 0.01) decrease in maximum survival time (Table 5, Figure 4d).

Pretreatment with −AWBE resulted in an 8% (*p* < 0.05) increase in male median survival time under hyperthermia, along with a right shift of the mortality curve (Table 5, Figure 4e). In females, when pretreated with −AWBE and +AWBE, the median survival time under hyperthermia increased by 33% (*p* < 0.05) and 50% (*p* < 0.05), respectively, and the mortality curves shifted to the right (Table 5, Figure 4f).

When comparing the effects of the pretreatment of *Drosophila* with −AWBE and +AWBE, it was revealed that no statistically significant differences were observed under conditions of oxidative stress (*p* > 0.05). Under conditions of starvation, the use of wheat bran extract containing anthocyanins resulted in a 12% (*p* < 0.01) increase in median survival time of females compared to those who consumed a wheat bran extract without anthocyanins. The +AWBE under hyperthermia decreased median male survival time by 23% (*p* < 0.05) compared to −AWBE (Table 5).

Thus, the effect of wheat bran extracts on stress resistance depends on sex, the type of stress factor, and the composition of the extract. The +AWBE, in most cases, has a more pronounced positive effect on stress resistance in females, especially during starvation. In males, the effect of both extracts on stress resistance is restricted.

## 4. Discussion

Aging is a complex biological process that is associated with the deterioration of the physiological functions of the body, as well as with the emergence of various age-dependent pathologies, which ultimately lead to death [67]. Although some studies suggest that consuming certain foods with antioxidants may improve health [5,68], the contribution of antioxidants to aging and longevity is controversial [23,69].

In the presented work, we studied the antioxidant properties, in vitro, and the geroprotective potential, in vivo, of −AWBE and +AWBE. It was found that both extracts showed high antioxidant potential. A higher RSA of grain bran extracts was also observed in anthocyanin-colored wheat lines compared to uncolored wheat lines in the study by Sharma, Yadav, Tiwari, Ali, Krishania, Bala, Mridula, Sharma, Goudar, Roy, Navik, and Garg [33]. The antioxidant activity of both whole wheat grain and bran extracts may be attributed to the presence of not only anthocyanins, but also other components, including a wide range of phenolic compounds [36,70,71,72]. Previously published data suggests that the RSA of extracts from the bran of different wheat varieties positively correlates with the total amount of phenolic compounds [73]. In particular, the high antioxidant activity of wheat bran extracts is associated with the presence of various hydroxycinnamic acids (mainly ferulic), lignans, phytic acid, alkylresorcinols, carotenoids, tocopherols, and tocotrienols [49,71,73,74,75,76,77]. The content of these compounds in wheat grain depends on both the genotype and growing conditions [71,73,76]. Individual fractions of polyphenols isolated from wheat bran, at concentrations of 0.02–0.03 mg/mL, were effective in reducing oxidative damage caused by H_2_O_2_ to Caco-2 cells [75].

It was previously revealed that wheat grains contain a substantial quantity of phenolic acids [41], which can be classified as hydroxybenzoic acids (gallic, *p*-hydroxybenzoic, vanillic, syringic, and protocatechuic acids) and hydroxycinnamic acids (caffeic, ferulic, and sinapic acids) [36]. Wheat bran contains a wide range of phenolic acids, including ferulic acid, *p*-coumaric acid, and sinapic acid, which exhibit antioxidant activity [41]. Also, depending on the wheat grain sample, HPLC analysis revealed the presence of phenolic acids and flavonoid components in the form of anthocyanins, including cyanidin-3-glucoside, peonidin-3-glucoside, and cyanidin-3-galactoside [36].

Some aspects of the biochemical composition of whole wheat flour extracts used in this study were previously published [43,78,79]. The lines exhibited no differences in protein, total dietary fiber content, total phenolic content (although the colored (referred here as anthocyanin-containing) line displayed a slight increase compared to the non-colored (referred here as anthocyanin-free) line, of which the difference was not statistically significant), metals, and amino acid content [78,79]. The anthocyanin levels in both whole grain and bran extracts were higher in the colored line compared to the non-colored line [43]. In accordance with a previously published study of Shamanin, Tekin-Cakmak, Gordeeva, Karasu, Pototskaya, Chursin, Pozherukova, Ozulku, Morgounov, Sagdic, and Koksel [79], the HPLC system detected gallic acid, protocatechuic acid, catechin, 4-hydroxybenzoic acid, syringic acid, ellagic acid, *m*-coumaric acid, *o*-coumaric acid, chrysin, caffeic acid, *p*-coumaric acid, ferulic acid, quercetin, kaempferol, rutin, sinapic acid, and chlorogenic acid in both anthocyanin-free and anthocyanin-containing extracts from the studied lines. Gallic acid, protocatechuic acid, and ellagic acid dominated the wheat samples of both anthocyanin-free and anthocyanin-containing lines and were detected in the free fractions. Thus, the high antioxidant potential of −AWBE and +AWBE may be associated with the presence of not only anthocyanins, but also other secondary metabolites of wheat.

We also studied the relationship between the antioxidant properties of wheat extracts and their geroprotective activity. We tested the hypothesis that, due to their antioxidant properties, wheat extracts may reduce free radical-induced intracellular damage and prolong the lifespan of *D. melanogaster*. Various previous studies have reported the geroprotective effect of various anthocyanin-rich wheat varieties on model organisms.

For example, the biofortified blue wheat, which contains approximately 110 mg/kg anthocyanins, lengthens the median lifespan of *D. melanogaster* by 26% compared to the control (white wheat flour) in different nutritional conditions [80]. The authors associate the results with a modulation of the expression of longevity genes, including *AMPK alpha* (an enzyme playing a crucial role in cell homeostasis), *SREBPs* (transcription factors that act as the primary regulators of lipid metabolism), *PEPCK* (an enzyme involved in gluconeogenesis), and *Cry* (a protein involved in the regulation of the circadian rhythm—the internal biological clock governing various physiological processes) [80]. Also, in the Chen et al. [81] study, purple wheat extract (*Triticum aestivum* L.), rich in anthocyanins, increased the average lifespan of wild-type nematodes (*Caenorhabditis elegans*) by 10.5% and *mev-1* mutants with an excessive production of free radicals by 9.2%, due to the activation of the transcription factor DAF-16/FOXO.

In support of our hypothesis and the previously published data from other authors, +AWBE increases the median lifespan (by 6%) of flies. However, the most significant increase in median lifespan (by 11%) and maximum lifespan (by 6%) was observed when treated with −AWBE. Our findings suggest that the increase in lifespan is linked to the antioxidant activity of the extracts. Moreover, antioxidant activity is associated with the presence in wheat extracts of not only anthocyanins, but also other phytochemicals found in the anthocyanin-free wheat extract [43,78,79]. This is consistent with the findings of Adom et al. [82], who showed that extracts of different varieties of wheat, along with flavonoids and ferulic acids, contain carotenoids, which can increase their antioxidant activity.

It is worth noting that the geroprotective effect of wheat bran extracts was most pronounced in females. Significant differences in male and female flies’ responses to nutritional supplements and drugs may be due to differences in their metabolic pathways, oxidative homeostasis, gut function, and responses to pharmacological treatment [83].

*D. melanogaster* is a suitable model for studying the effects of geroprotective interventions on age-related alterations in different physiological functions, due to its similarities to human functional aging, including a reduction in locomotor activity with age [17,59,60]. Although there is no significant correlation between the number of movements of flies and their potential lifespan [84]. Oxidative damage has been linked to numerous diseases and conditions, including a decline in physical activity. For example, a study utilizing a mouse model with elevated oxidative stress and neurogenic atrophy discovered that an increase in free radical production was associated with decreased physical activity [85]. Free radicals can also cause oxidative damage to proteins, membrane lipids, and DNA, potentially contributing to the progression of amyotrophic lateral sclerosis [86]. Accordingly, an increase in antioxidant protection can contribute not only to an increase in lifespan, but also to an improvement in its quality. In this study, +AWBE increased locomotor activity in males. This finding is consistent with a study previously published by Li et al. [87], which showed that the treatment of *D. melanogaster* individuals with anthocyanin extract from black rice (*Oryza sativa* L.) resulted in a postponed decrease in motor function. There are also notable sex differences in age-related changes in motor activity. Motor activity in females consistently decreases with age, while in males, it increases until the age of five weeks, before eventually decreasing. Our results on age-related changes in the locomotor activity of males and females are consistent with the literature data [60].

The molecular mechanisms that regulate lifespan and cellular response to adverse factors, including the antioxidant defense system, heat shock proteins, and the insulin/IGF-like signaling pathway, are interconnected [88,89], which underlies the correlation between stress resistance and longevity [90]. Resistance to stress factors reflects the physiological condition of an organism and is linked to its lifespan [91]. Generally, *Drosophila* that live longer exhibit greater resistance to adverse environmental conditions [90], including oxidative stress [92]. Since numerous geroprotective compounds possess antioxidant activity and enhance stress resistance [93], we evaluated the impact of wheat bran extracts on the survival of flies exposed to oxidative stress, starvation, and hyperthermia.

Previous research has demonstrated the effectiveness of blue wheat diets in enhancing the survival rates of flies with suppressed superoxide dismutase 2 (SOD2), as well as increasing resistance to oxidative stress induced by paraquat via the consumption of diets based on blue and purple wheat varieties [80]. Despite the established antioxidant activity of wheat extracts in vitro, our study did not uncover a significant effect of −AWBE and +AWBE on flies’ resistance to oxidative stress caused by the prooxidant paraquat. It is possible that the protective effect of the extracts may occur at lower concentrations of paraquat, but further research is necessary to confirm this.

At the same time, treatment with −AWBE and +AWBE led to increased resistance to hyperthermia in *D. melanogaster* of both sexes. It is known that elevated temperatures disrupt metabolic processes, accentuating the accumulation of damage in the body and therefore hastening the organism’s death [94]. It is possible that the wheat bran extracts we studied activated protective mechanisms, due to the expression of heat shock genes, such as *hsp70*, that are necessary for survival in unfavorable environmental conditions [95]. For example, the intake of Sunrouge (*Camellia taliensis* × *C. sinensis* L.) tea extract enriched with anthocyanins significantly increased the expression of HSP40, HSP70, and HSP32 in the intestines of mice [96].

Under conditions of starvation, −AWBE and +AWBE reduced survival of both male and female flies. It is assumed that a model organism subjected to starvation conditions can develop adaptive plasticity in response to this stressor via alterations in carbohydrate and lipid metabolism, lipid storage, potential impairment of reproductive function, and a general reduction in energy expenditure, which increases survival likelihood [97]. However, the role of anthocyanins in this process has not yet been sufficiently studied. At the same time, it is obvious that the samples from anthocyanin-free and anthocyanin-containing wheats do not contain sufficient concentrations of lipids and carbohydrates that could improve the adaptation of *D. melanogaster* to conditions of starvation. A previously studied ethanol extract derived from chokeberry (×*Sorbaronia mitschurinii*) also did not demonstrate a statistically significant impact on resistance to oxidative stress and hyperthermia, but it increased the survival time of *D. melanogaster* females under conditions of starvation [25].

When applying rowan (*Sorbus aucuparia*) fruit extract to examine the survival of fruit flies in unfavorable environmental conditions, a notable reduction in female survival rates was observed under oxidative stress conditions [27]. The extract of rowan berry, on the other hand, increased male resistance in starvation conditions while reducing resistance to hyperthermia. Under hyperthermic conditions, the administration of rowan berry extract was observed to enhance survival rates in female subjects. In a study utilizing honeysuckle extract (*Lonicera pallasii* L.) and cyanidin-3-glucoside, a significant improvement in the survival rate of *Drosophila* males under oxidative stress conditions was observed [26]. These findings suggest that the existence of anthocyanins in diet may not always be the predominant factor in controlling the redox process, which in turn affects the aging of the body.

## 5. Conclusions

In recent years, wheat breeding has been focused on accumulating anthocyanin compounds in the grain, which can improve food quality and human health [33,34,35]. Previous studies have revealed that anthocyanins remain stable during bread production [43,44] and can be easily incorporated into human diet by using purple grain in bakery products [42].

The present study investigates the antioxidant and geroprotective properties of wheat bran extracts utilizing both in vitro and in vivo research methods. HPLC-MS demonstrated the presence of cyanidin glucoside and cyanidin arabinoside in the bran extract of the anthocyanin-containing wheat line, whereas no anthocyanins were detected in the bran extract of the anthocyanin-free wheat line.

When assessing the antioxidant activity of extracts, different test systems were used, including mammalian erythrocytes and substrates based on tissue homogenates of laboratory animals. It was found that the bran extract from the anthocyanin-containing wheat was characterized by a higher radical scavenging activity (DPPH and ABTS assays) as well as a greater membrane protective activity in the model of AAPH-oxidative hemolysis, compared to the bran extract from the anthocyanin-free wheat. Both extracts were effective in reducing lipid peroxidation initiated by the Fe^2+^/ascorbate and H_2_O_2_ in a heterogeneous substrate (oil in water emulsions) derived from brain and testes tissue homogenates. The extracts also showed a reduction in H_2_O_2_-induced oxidative hemolysis in erythrocytes of laboratory animals. However, no significant differences in the effectiveness between extracts were observed in these experiments.

To study the effects of wheat bran extracts on living organisms, *D. melanogaster* was utilized as the model system. Both anthocyanin-free and anthocyanin-containing wheat bran extracts demonstrated a lifespan-extending effect in *Drosophila* females.

Thus, our study demonstrates that wheat bran extracts containing high levels of anthocyanins have antioxidant and geroprotective effects. However, the obtained results suggest that other secondary metabolites in wheat bran can also contribute to its antioxidant and geroprotective potential.

Further studies on the benefits of wheat bran can assist in developing strategies aimed at promoting healthy longevity and enhancing the quality of human life via healthy and functional nutrition.

## Figures and Tables

**Table 1:** Comparative evaluation of AOA * (test on the substrate from the brain and testes) * and RSA (tests with DPPH and ABTS) of the bran extracts.

Variant	RSA (DPPH), %	RSA (ABTS), %	TBA-RS *, nmol/mL	
			Brain Substrate	Testes Substrate
Control	–	–	55.0 ± 0.5	16.6 ± 0.3
Intact	–	–	36.6 ± 0.3	9.9 ± 0.1
Extract concentration is 0.5 mg/mL				
−AWBE	12.5 ± 0.1	57.3 ± 0.3	5.0 ± 0.2	8.7 ± 0.3
+AWBE	24.3 ± 0.5 *	82.4 ± 0.3 *	5.2 ± 0.2	9.4 ± 0.3
Trolox	96.0 ± 0.0	99.4 ± 0.0	4.4 ± 0.0	4.9 ± 0.1
Extract concentration is 0.05 mg/mL				
−AWBE	3.2 ± 0.3	30.9 ± 0.3	6.8 ± 0.1	–
+AWBE	4.3 ± 0.1	47.3 ± 0,1	6.2 ± 0.4	–
Trolox	96.7 ± 0.0	99.4 ± 0.0	4.5 ± 0.0	–

* The extract’s ability to inhibit the accumulation of TBA-RS was evaluated in substrates obtained from mice tissue homogenates (brain and testes), 1 h after initiation of LPO with Fe^2+^/ascorbate (brain) or 3 h after initiation of LPO with H_2_O_2_ (testes). Control is sample without the tested extract in which LPO was initiated; intact is sample without the extract in which LPO was not initiated. Experimental data are presented as mean ± SE (*n* = 4). +AWBE and −AWBE variants were compared using the Mann–Whitney test: * *p* < 0.05.

**Table 2:** Membrane protective activity of bran extracts at a concentration of 0.05 mg/mL. The level of H_2_O_2_- and AAPH-induced hemolysis after 1–5 h of cell incubation is presented.

Sample	Hemolysis, %				
	1 h	2 h	3 h	4 h	5 h
H_2_O_2_					
Control	18.7 ± 0.7	32.3 ± 0.8	39.2 ± 0.5	45.9 ± 0.6	50.5 ± 0.6
−AWBE	11.2 ± 0.4	22.4 ± 0.8	32.4 ± 0.9	40.7 ± 0.7	45.7 ± 0.5
+AWBE	11.8 ± 0.6	23.6 ± 0.6	34.6 ± 0.3	41.7 ± 0.4	45.6 ± 0.3
Trolox	5.5 ± 0.1	8.8 ± 0.2	9.3 ± 0.2	9.3 ± 0.2	9.3 ± 0.2
AAPH					
Control	1.7 ± 0.2	9.8 ± 1.0	55.6 ± 1.8	77.5 ± 0.4	84.9 ± 0.5
−AWBE	3.5 ± 0.1	4.7 ± 0.2	22.7 ± 2.2	72.1 ± 0.5	85.4 ± 0.3
+AWBE	3.1 ± 0.3	4.5 ± 0.3	10.4 ± 2.1 **	52.4 ± 1.6 **	79.6 ± 1.1 **
Trolox	2.7 ± 0.1	3.7 ± 0.1	4.5 ± 0.1	5.2 ± 0.3	6.3 ± 0.3

Experimental data are presented as mean ± SE (*n* = 4). +AWBE and −AWBE variants were compared using the Mann–Whitney test: ** *p* < 0.01.

**Table 3:** Effect of −AWBE and +AWBE on the lifespan of *D. melanogaster*.

Variant	Sex	M			90%			LR, *p*	N
		M, Days	dM, % (CTR)	dM, % (ACN)	90%, Days	d90%, % (CTR)	d90%, % (ACN)		
Control	♂	60	n/a	n/a	67	n/a	n/a	n/a	601
−AWBE	♂	60	0	n/a	67	0	n/a	>0.05	585
+AWBE	♂	57	−5 ***	−5	67	0	0	<0.05	573
Control	♀	54	n/a	n/a	67	n/a	n/a	n/a	586
−AWBE	♀	60	+11 ***	n/a	71	+6 **	n/a	<0.001	577
+AWBE	♀	57	+6 ***	−5 ***	68	+1	−4 ***	<0.05	592

♂—males; ♀—females; M, days—median lifespan; 90%, days—age of 90% mortality; dM, % and d90%, %—differences in median lifespan and age of 90% mortality, respectively; (CTR)—comparison of −AWBE and +AWBE variants with the control variant; (ACN)—comparison of −AWBE and +AWBE variants; LR, *p*—log-rank test with Bonferroni correction *p*-value; n/a—not applicable; N—number of individuals in the sample; ** *p* < 0.01, *** *p* < 0.001—Fisher’s exact test with Bonferroni correction.

**Table 4:** Results of a two-way ANOVA for the evaluation of the effect of age and treatment on the locomotor activity of *D. melanogaster*.

Source of Variation	SS	DF	MS	F	*p*
males					
Age	3.93 × 10^9^	7	5.62 × 10^8^	22.638	7.9 × 10^−18^
Treatment	2.16 × 10^8^	2	1.08 × 10^8^	4.358	0.0154
Age × Treatment	8.7 × 10^8^	14	62,150,972	2.505	0.0044
Error	2.38 × 10^9^	96	24,809,477		
females					
Age	5.94 × 10^8^	7	84,872,011	76.314	2.1 × 10^−36^
Treatment	9,096,658	2	4,548,329	4.09	0.0197
Age × Treatment	12,545,984	14	896,141.8	0.806	0.6609
Error	1.07 × 10^8^	96	1,112,145		

SS—sum of squared deviations; DF—number of degrees of freedom; MS—dispersion; F—actual value of the Fisher ratio. *p*—*p*-value.

**Table 5:** The influence of −AWBE and +AWBE on the survival time of *D. melanogaster* under stressful conditions.

Variant	Sex	M			90%			LR, *p*	N
		M, h	dM, % (CTR)	dM, % (ACN)	90%, h	d90%, % (CTR)	d90%, % (ACN)		
Oxidative stress									
Control	♂	67	n/a	n/a	89	n/a	n/a	n/a	96
−AWBE	♂	64	−4	n/a	93	+4	n/a	>0.05	96
+AWBE	♂	69	+3	+8	91	+2	−2	>0.05	96
Control	♀	66	n/a	n/a	97	n/a	n/a	n/a	96
−AWBE	♀	66	0	n/a	90	−7	n/a	>0.05	95
+AWBE	♀	59	−11	−11	85	−12	−6	>0.05	64
Starvation									
Control	♂	53	n/a	n/a	72	n/a	n/a	n/a	96
−AWBE	♂	48	−9 **	n/a	68	−6	n/a	<0.05	96
+AWBE	♂	47	−11 ***	−2	69	−4	+1	<0.05	96
Control	♀	65	n/a	n/a	122	n/a	n/a	n/a	96
−AWBE	♀	68	+5	n/a	99	−19 **	n/a	>0.05	96
+AWBE	♀	76	+17	+12 **	106	−13	+7	>0.05	96
Hyperthermia									
Control	♂	12	n/a	n/a	15	n/a	n/a	n/a	64
−AWBE	♂	13	+8 *	n/a	17	+13	n/a	<0.05	64
+AWBE	♂	10	−17	−23 *	15	0	−12	>0.05	64
Control	♀	6	n/a	n/a	12	n/a	n/a	n/a	64
−AWBE	♀	8	+33 *	n/a	12	0	n/a	<0.05	64
+AWBE	♀	9	+50 *	+13	13	+8	+8	<0.01	64

♂—males; ♀—females; M, h—median survival time; 90%, h—maximum survival time; dM, %, and d90%; %—differences in median and maximum survival time, respectively; (CTR)—comparison of −AWBE and +AWBE variants with the control variant; (ACN)—comparison of −AWBE and +AWBE variants; LR, *p*—log-rank test with Bonferroni correction *p*-value; n/a—not applicable; N—number of individuals in the sample; * *p* < 0.05, ** *p* < 0.01, *** *p* < 0.001—Fisher’s exact test with Bonferroni correction.

## Data Availability

All data generated or analyzed during this study are included in this published article.

## Supplementary Materials

The following supporting information can be downloaded at: https://www.mdpi.com/article/10.3390/antiox12112010/s1, Figure S1: Calibration graph of cyanidin glucoside; Table S1: Retention time, concentration, and molecular ion of major and minor anthocyanins in the extraction mixture.

## Abbreviations

+AWBE: anthocyanin-containing wheat bran extract; −AWBE: anthocyanin-free wheat bran extract; AAPH: 2:2′-azobis(amidinopropane) dihydrochloride; ABTS: 2,2′-azino-bis-(3-ethylbenzthiozoline-6-sulfonic acid) diammonium salt; AcN: acetonitrile; AOA: antioxidant activity; RSA: radical scavenging activity; DPPH: 2,2-diphenyl-1-picrylhydrazyl; H_2_O: water; H_2_O_2_: hydrogen peroxide; HCl: hydrochloric acid; HCOOH: formic acid; HPLC-MC: high-performance liquid chromatography-mass spectrometry; LPO: lipid peroxidation; TBA: 2-thiobrabituric acid; TBA-RS: TBA-reactive substances.
