# Supplementary material for: Antioxidant Properties and Geroprotective Potential of Wheat Bran Extracts with Increased Content of Anthocyanins
Source: Antioxidants (Basel). 2023 Nov 17;12(11):2010. doi: 10.3390/antiox12112010 (PMC10669849; doi:10.3390/antiox12112010)
Supplement: Supplementary file 1 [file antioxidants-12-02010-s001.zip › Table S1.pdf]

Table S1. Retention time, concentration and molecular ion of major and minor anthocyanins in the extraction mixture.

Anthocyanins	Retention time, min	Peak area	Concentration, mg/ml	m/z
Major components				
Cyanidin glucoside	2.96	54092	0.054	486.21
Cyanidin arabinoside	6.60	57480	0.057	426.33
Minor components				
Cyanidin galactoside	2.23	3611	0.0036	485.96
Pelargonidin glucoside	4.58	5613	0.0056	450.03
Malvidin glucoside	7.92	6067	0.0060	510.42
