# Supplementary figures and images for: Antioxidant Properties and Geroprotective Potential of Wheat Bran Extracts with Increased Content of Anthocyanins
Source: Antioxidants (Basel). 2023 Nov 17;12(11):2010. doi: 10.3390/antiox12112010 (PMC10669849; doi:10.3390/antiox12112010)

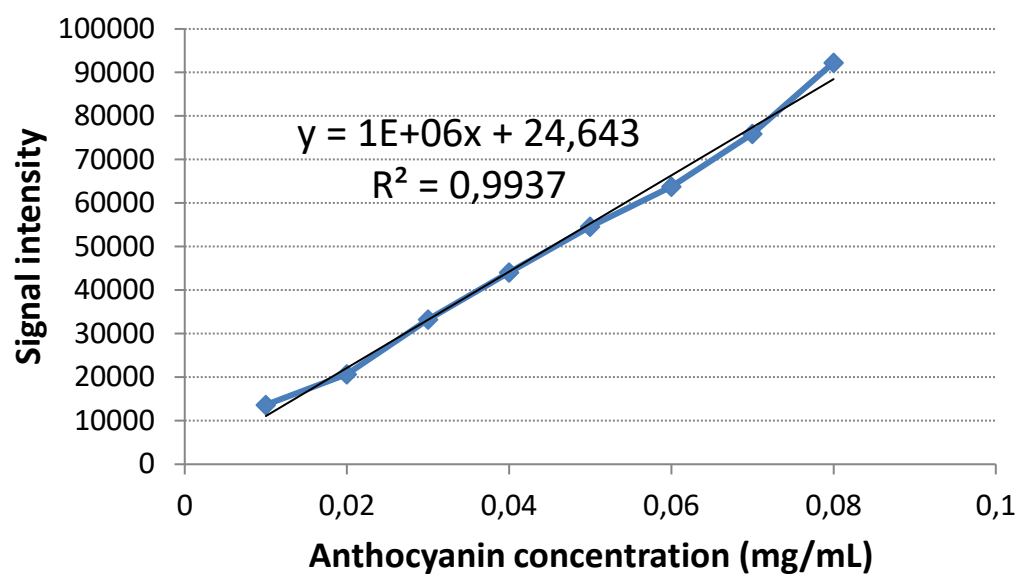

**Figure S1.** Calibration graph of cyanidin glucoside.

Supplement: Supplementary file 1 [file antioxidants-12-02010-s001.zip › Figure S1.pdf]
